# Pancreatic Volume in Thalassemia: Determinants and Association with Alterations of Glucose Metabolism

**DOI:** 10.3390/diagnostics15050568

**Published:** 2025-02-26

**Authors:** Antonella Meloni, Gennaro Restaino, Vincenzo Positano, Laura Pistoia, Petra Keilberg, Michele Santodirocco, Anna Spasiano, Tommaso Casini, Marilena Serra, Emanuela De Marco, Maria Grazia Roberti, Sergio Bagnato, Alessia Pepe, Alberto Clemente, Massimiliano Missere

**Affiliations:** 1 Bioengineering Unit, Fondazione G. Monasterio CNR-Regione Toscana, 56124 Pisa, Italy; positano@ftgm.it; 2 Radiology Department, Responsible Research Hospital, 86100 Campobasso, Italy; gennaro.restaino@gemellimolise.it (G.R.); massimiliano.missere@gemellimolise.it (M.M.); 3 Unità Operativa Complessa Ricerca Clinica, Fondazione G. Monasterio CNR-Regione Toscana, 56124 Pisa, Italy; laura.pistoia@ftgm.it; 4 Department of Radiology, Fondazione G. Monasterio CNR-Regione Toscana, 56124 Pisa, Italy; petra.keilberg@ftgm.it; 5 Centro Microcitemia—Day Hospital Thalassemia Poliambulatorio “Giovanni Paolo II”, Ospedale Casa Sollievo della Sofferenza IRCCS, 71013 San Giovanni Rotondo, Italy; m.santodirocco@operapadrepio.it; 6 Unità Operativa Semplice Dipartimentale Malattie Rare del Globulo Rosso, Azienda Ospedaliera di Rilievo Nazionale “A. Cardarelli”, 80131 Napoli, Italy; spasiano.anna@tiscali.it; 7 SOC Oncologia, Ematologia e Trapianto di Cellule Staminali Emopoietiche, Meyer Children’s Hospital IRCCS, 50139 Firenze, Italy; tommaso.casini@uslcentro.toscana.it; 8 Day Hospital di Talassemia, Ospedale “V. Fazzi”, 73100 Lecce, Italy; marilenaserra@libero.it; 9 Unità Operativa Oncoematologia Pediatrica, Azienda Ospedaliero Universitaria Pisana—Stabilimento S. Chiara, 56126 Pisa, Italy; e.demarco@ao-pisa.toscana.it; 10 Servizio Trasfusionale, Azienda Ospedaliero-Universitaria OO.RR. Foggia, 71100 Foggia, Italy; mroberti@ospedaliriunitifoggia.it; 11 Ematologia Microcitemia, Ospedale San Giovanni di Dio—ASP Crotone, 88900 Crotone, Italy; krthal@libero.it; 12 Institute of Radiology, Department of Medicine, University of Padua, 35128 Padua, Italy; alessia.pepe@unipd.it

**Keywords:** pancreatic volume, magnetic resonance imaging, thalassemia, iron overload, glucose metabolism

## Abstract

**Objectives:** This study aimed to compare the pancreatic volume between beta-thalassemia major (β-TM) and beta-thalassemia intermedia (β-TI) patients and between thalassemia patients and healthy subjects and to determine the predictors of pancreatic volume and its association with glucose metabolism in β-TM and β-TI patients. **Methods:** We considered 145 β-TM patients and 19 β-TI patients enrolled in the E-MIOT project and 20 healthy subjects. The pancreatic volume and pancreatic and hepatic iron levels were quantified by magnetic resonance imaging. **Results:** The pancreatic volume indexed by body surface area (PVI) was significantly lower in both β-TI and β-TM patients compared to healthy subjects and in β-TM patients compared to β-TI patients. The only independent determinants of PVI were pancreatic iron in β-TM and hepatic iron in β-TI. In β-TM, there was an association between alterations of glucose metabolism and PVI, and PVI was a comparable predictor of altered glucose metabolism compared to pancreatic iron. Only one β-TI patient had an altered glucose metabolism and showed a reduced PVI and pancreatic iron overload. **Conclusions:** Thalassemia syndromes are characterized by a reduced pancreatic volume, associated with iron levels. In β-TM, the pancreatic volume and iron deposition are associated with the development and progression of alterations of glucose metabolism.

## 1. Introduction

Beta-thalassemia is an inherited blood disorder caused by impaired beta globin chain synthesis, leading to ineffective erythropoiesis and chronic anemia [1,2]. Beta-thalassemia major (β-TM) is the most severe form, and affected individuals require regular blood transfusions from early childhood to maintain adequate hemoglobin levels [3,4]. Despite its pivotal role in treating anemia, repeated transfusions lead to the progressive accumulation of iron in various tissues, including the liver, heart, and endocrine organs, such as the pancreas [5,6,7]. Beta-thalassemia intermedia (β-TI) lies on a spectrum of disease severity between TM and thalassemia minor (asymptomatic carrier). Some β-TI patients can remain asymptomatic for most of their lives, presenting with moderate anemia, whereas others, at some stage of their illness, necessitate regular blood transfusions to prevent or handle complications associated with chronic anemia [8,9,10,11,12]. In β-TI, iron overload can also occur in the absence of transfusions due to the ineffective erythropoiesis, which triggers the suppression of hepcidin synthesis, leading to the upregulation of intestinal iron absorption and iron release from macrophages [13,14]. The prevalence of extra-hepatic iron overload has been demonstrated to increase with the administration of regular transfusions [15,16]. Anyway, in regularly transfused β-TI patients, pancreatic iron deposition occurs at a much slower rate than in β-TM patients [17,18], likely due to the reduced duration and intensity of the transfusional regimen.

Pancreatic iron deposition initiates a cascade of events characterized by oxidative stress, mitochondrial dysfunction, and, ultimately, pancreatic beta-cell injury and apoptosis. This sequence of pathological changes culminates in impaired insulin secretion and the eventual onset of diabetes mellitus (DM) [19,20,21]. According to the literature, β-TM is characterized by an increased prevalence of diabetes compared to β-TI [22,23], likely attributable to the greater extent of pancreatic iron loading [15,17,18]. Anyway, it is important to underline that, in thalassemia, glucose dysregulation is a complex process influenced by multiple factors. In addition to impaired insulin secretion, insulin resistance also plays a significant role. This resistance arises from hepatic iron overload, which disrupts insulin’s ability to suppress glucose production, and iron accumulation in muscle tissue, which reduces glucose uptake [24].

Several studies conducted on non-thalassemic populations found that the pancreatic volume was significantly reduced in individuals with diabetes compared to normoglycemic subjects [25,26,27,28,29]. In DM, pancreatic atrophy, characterized by the almost complete disappearance of the acinar cells and of the islets of Langerhans to a lesser extent, has been linked to the effects of chronic inflammation [30,31] and the loss of insulin’s trophic effects on the exocrine pancreas [26,30,31]. On the other side, it has been suggested that a reduced baseline pancreatic volume could contribute to the pathophysiology of diabetes, potentially due to a lower reserve of functional pancreatic tissue or impaired beta-cell mass [27,32]. Of note, a low birth weight is a recognized risk factor for type 2 DM [33]. This relationship may be linked to a smaller pancreatic size, supporting the hypothesis of a lower pancreatic volume as a predisposing factor for the development of DM. Anyway, whether a decreased pancreatic size is a risk factor or a downstream result of diabetes is still an open question [28].

The pancreatic volume and its clinical correlations in thalassemia have been little explored. Au et al., in a cohort of 72 β-TM patients, found that diabetes was associated with a significantly reduced pancreatic volume. However, they failed to detect an association between pancreatic volume and iron levels [34]. To the best of our knowledge, no study has evaluated the pancreatic volume in β-TI. The results observed in β-TM patients cannot be directly extrapolated to β-TI due to differences in molecular mechanisms and treatment strategies, such as transfusion and iron chelation.

The purposes of this observational, cross-sectional study were (1) to compare pancreatic volume between β-TM and β-TI patients and between thalassemia patients and healthy subjects; (2) to determine the predictors of pancreatic volume in β-TM and β-TI patients; (3) to assess the association between pancreatic volume and glucose metabolism β-TM and β-TI patients.

## 2. Materials and Methods

### 2.1. Study Population

The Extension-Myocardial Iron Overload in Thalassemia (E-MIOT) project is an Italian network including 66 thalassemia centers and 15 magnetic resonance imaging (MRI) sites where MRI exams are performed using homogeneous, standardized, and validated procedures [35,36]. All centers are linked by a shared web-based database gathering demographic, clinical, laboratory, and instrumental data. The inclusion criteria of the E-MIOT project are (1) female and male patients of all ages, with genetically diagnosed thalassemia syndromes or structural hemoglobin variants, requiring T2* MRI to quantify cardiac, hepatic, and pancreatic iron levels; (2) written informed consent; (3) written authorization for use and disclosure of protected health information; (4) no absolute contraindications to MRI. In two MRI centers of the E-MIOT Network (Pisa and Campobasso), the sequence for the quantification of pancreatic volume was added to the standard imaging protocol (multi-organ T2* + assessment of cardiac function) starting from September 2016. We included in this study all 159 β-TM patients (60.4% females; 38.91 ± 10.01 years) and 19 β-TI patients (52.6% females; 39.63 ± 12.67 years) consecutively enrolled in the E-MIOT project and prospectively scanned with the “expanded” MRI protocol from September 2016 to June 2020.

Moreover, we included 20 healthy subjects (50% females; 54.03 ± 15.19 years) without pancreatic diseases, systemic diseases, or alterations in glucose metabolism.

The study complied with the Declaration of Helsinki and was approved by the ethical committees of all MRI sites. All subjects gave written informed consent.

### 2.2. MRI

MRI was performed using a 1.5-T scanner (Signa Excite HD or Signa Artist, GE Healthcare, Milwaukee, WI, USA). Patients were scanned in the supine position with a phased-array receiver surface coil.

A coronal localizer image was used to assess the topography of the pancreas. No contrast media were administered.

Axial slices covering the entire pancreas were acquired by a T1-weighted gradient echo (GRE) in-phase and out-phase sequence. Imaging parameters were as follows: slice thickness 8 mm, flip angle 85°, matrix 512 × 512 pixels, field of view 38 × 30 cm, echo times (TEs) 2.26 and 4.52 ms, and repetition time (TR) 150 ms.

A mid-transverse hepatic slice [37] and five or more axial slices, including the whole pancreas [38], were acquired with a T2* multiecho GRE sequence for iron overload assessment. Each single slice was acquired in a single end-expiratory breath-hold at 10 TEs with an echo spacing of 2.26 ms. The scan time for each breath hold was about 12 s. For both organs, the multiecho sequence parameters were as follows: slice thickness 8 mm, flip angle 25°, matrix 192 × 192 pixels, bandwidth 62.5 KHz, and TR 34 ms. The minimum possible TE was used for the liver, while the first TE for the pancreas was 2.0 ms.

Image analysis was performed by expert operators with more than 15 years of experience in abdominal MRI, using a custom-written, previously validated software (HIPPO MIOT^®^, Version 2.0, Consiglio Nazionale delle Ricerche and Fondazione Toscana Gabriele Monasterio, Pisa, Italy, Year 2015).

**Figure 1:**
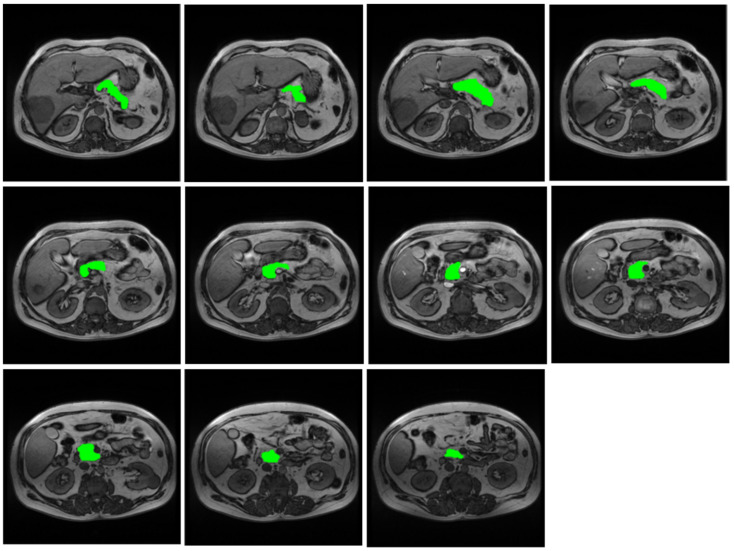
Procedure for the evaluation of pancreatic volume from axial T1-weighted GRE MRI images. A region of interest encompassing the pancreatic parenchyma was manually traced in each slice and its cross-sectional area was calculated. All such measurements through the pancreas were multiplied by the slice thickness and summed to calculate the pancreatic volume.

In-phase T1-weighted GRE images were used for pancreatic volume assessment. The pancreatic parenchyma was manually outlined using a free-hand region of interest (ROI) on all slices where the pancreas was visible (Figure 1). Great care was taken to accurately contour each lobulation of the pancreas and to exclude surrounding fat, the common bile duct, and the pancreatic duct. The pancreatic area on each slice was automatically determined. The pancreatic volume was computed using the Simpson method: all cross-sectional areas were multiplied by the slice thickness and summed [39]. The approximate time needed to generate the pancreatic volume was 10 min, although this varied depending on the number of slices and the contour variations in the pancreas. To eliminate the effect of the subject’s body size on pancreatic size while retaining correlation to the original measurement and to enable accurate comparisons, pancreatic volume was indexed by the body surface area (BSA), derived using the variation in the Dubois and Dubois formula [40].

**Figure 2:**
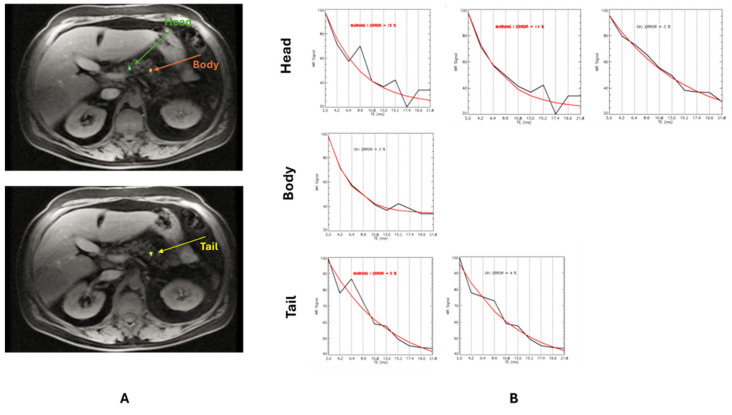
Procedure for the evaluation of the T2* value in the pancreas from multiecho MRI images. (**A**) Three small ROIs were traced in head, body, and tail regions on the image with the best contrast (TE = 4.2 ms in the illustrated case). (**B**) The extracted signal-to-TE curve was fitted to an appropriate decay model to estimate the T2* value in the ROI. In case of high fitting error (i.e., >5%), the signal corresponding to the TEs with the greater signal deviation were progressively excluded until an acceptable fitting error was reached.

For the T2* assessment, a region-based approach was employed. A circular ROI of standard dimension was drawn in an area of homogeneous liver tissue, avoiding blood vessels and other sources of artifacts [37]. In the pancreatic parenchyma, three small ROIs were hand-traced over the head, body, and tail, avoiding confounding anatomy (e.g., large blood vessels or ducts) and areas affected by susceptibility artifacts from gastric or colic intraluminal gas [41] (Figure 2A). For each ROI, the mean value of the signal intensity along all TEs was measured. The obtained decay curve was fit to a single exponential with a constant offset model:

(1) $$S=S_{0}e^{-\frac{TE}{T2*}}+C$$

where S represents the mean signal intensity, S_0_ is the signal intensity at TE = 0, T2* is the relaxation time, TE represents the echo times, and C is a constant value that takes into account the rectification of MRI noise. Decay curve fitting was performed by the Levenberg–Marquadt method, and the T2* value for the ROI was obtained. For the pancreas, in case of high fitting errors (>5%) caused by the presence of severe fatty infiltration, the signal corresponding to the TEs with the greater deviation from the exponential decay was progressively excluded until an acceptable fitting error was reached (Figure 2B) [41]. Hepatic T2* values were converted into liver iron concentration (LIC) values using Wood’s calibration curve [42,43]. The global pancreatic T2* value was calculated by averaging the T2* values from the three regions.

### 2.3. Biochemical Investigations

All biochemical investigations were performed using commercially available kits at the laboratories of thalassemia centers where the patients were treated.

The average value of hemoglobin and ferritin levels over the last 12 months before the MRI was considered.

To assess the disturbances of glucose metabolism, patients not already diagnosed with diabetes performed an oral glucose tolerance test (OGTT) within three months of the MRI study. Baseline (after overnight fasting) blood assessments of glucose were performed. Patients were given 1.75 g/kg (maximum dose = 75 g) of glucose solution, and glucose was assessed at 60 and 120 min.

### 2.4. Diagnostic Criteria

The lower limit of BSA-corrected pancreatic volume in healthy individuals was defined as mean–2 standard deviations (SD).

The lowest threshold of normal T2* pancreatic value was 26 ms [38].

A fasting plasma glucose (FPG) < 100 mg/dL and 2 h glucose < 140 mg/dL was considered normal glucose tolerance (NGT). Impaired fasting glucose (IFG) was diagnosed in the presence of FPG levels between 100 and 126 mg/dL. Impaired glucose tolerance (IGT) was defined by 2 h plasma glucose between 140 and 200 mg/dL, with a FPG < 126 mg/dL. DM was defined by FPG ≥ 126 mg/dL or 2 h glucose ≥ 200 mg/dL during an OGTT or random plasma glucose ≥ 200 mg/dL with classic symptoms of hyperglycemia [44].

### 2.5. Statistical Analysis

Statistical analyses were performed using MedCalc version 19.8 (MedCalc Software Ltd., Ostend, Belgium) and SPSS version 27.0 (IBM Corp., Armonk, NY, USA) statistical packages.

Continuous variables were represented by mean and standard deviation. Categorical variables were represented by absolute frequency and percentage.

The Kolmogorov–Smirnov test was used to assess the normality of the distribution of the parameters. Shapiro–Wilk test was used for a sample size ≤50.

Correlation analyses were performed using Pearson’s test or Spearman’s test as appropriate with respect to data distribution.

For quantitative data, the comparison between the two groups was made using either the student *t*-test or the Mann–Whitney test (non-parametric *t*-test) as appropriate. The comparison of categorical data was performed using Pearson’s Chi-square or Fisher’s exact tests.

The analysis of covariance (ANCOVA) was used to evaluate whether between-group differences persisted after controlling for potential covariates. Covariates were included if they significantly differed between groups and were associated with the assessed outcome. When necessary, outcomes were log-transformed to normalize the residual distributions and to equalize the residual variance.

Univariate and multivariate (stepwise forward) linear regression models were used to identify the determinants of pancreatic volume. Only significant variables from the univariate analysis were included in the multivariable tests. The collinearity of variables included in the multivariate model was investigated through the variance inflation factor (inflated if > 5) and the tolerance statistic (inflated if < 0.20).

Logistic regression evaluated the odds ratio (OR) with 95% confidence intervals (CI). The OR was used to compare the odds for the two groups.

The receiver operating characteristic (ROC) analysis was applied to estimate the diagnostic value of pancreatic size and iron levels for discriminating the presence of an altered OGTT. The results were presented as areas under the curve (AUCs) with 95% confidence intervals, and the optimal cut-off values were calculated using the Youden index method. The DeLong test was employed to compare the differences between the ROC curves.

In all tests, a 2-sided *p*-value < 0.05 was classified as statistically significant.

## 3. Results

### 3.1. Derivation of the Patient Population

MRI revealed an atrophic pancreas with complete fatty infiltration and no detectable normal pancreatic parenchyma in 14 (8.8%) β-TM patients. If compared to the other β-TM patients, these 14 patients were significantly older (46.65 ± 4.72 years vs. 38.16 ± 10.78 years; *p* < 0.0001) and more frequently females (92.9% vs. 57.2%; *p* = 0.009). Due to the impossibility of accurately measuring pancreatic volume, the 14 patients with complete fatty infiltration were excluded from the study. The demographic and clinical characteristics of the considered β-TM (*N* = 145) and β-TI (*N* = 19) patients are summarized in Table 1.

### 3.2. Association Between Pancreatic Volume and Gender in Thalassemia

Males exhibited a significantly higher BSA than females in both the β-TM group (1.69 ± 0.19 m^2^ vs. 1.54 ± 0.13 m^2^; *p* < 0.0001) and the β-TI group (1.82 ± 0.09 m^2^ vs. 1.57 ± 0.16 m^2^; *p* = 0.001). In β-TM, pancreatic volume was significantly higher in males than in females (46.75 ± 26.19 cm^3^ vs. 36.58 ± 17.24 cm^3^ *p* = 0.014), while the BSA-corrected pancreatic volume was comparable between the two sexes (27.55 ± 14.94 cm^3^/m^2^ vs. 23.94 ± 11.72 cm^3^/m^2^, *p* = 0.197). In β-TI, pancreatic volume was significantly higher in males than in females (66.25 ± 20.11 cm^3^ vs. 44.65 ± 17.76 cm^3^, *p* = 0.024), while the BSA-corrected pancreatic volume was comparable between the two sexes (36.63 ± 11.73 cm^3^/m^2^ vs. 28.60 ± 11.21 cm^3^/m^2^, *p* = 0.146).

### 3.3. Comparison Between Thalassemia Patients and Healthy Individuals

In healthy subjects, the BSA-indexed pancreatic volume was comparable between males and females (46.58 ± 3.34 cm^3^/m^2^ vs. 49.99 ± 6.27 cm^3^/m^2^, *p* = 0.315) and was not correlated with aging (R = −0.248, *p* = 0.489). The mean BSA-indexed pancreatic volume in healthy subjects was 48.28 ± 5.07 cm^3^/m^2^ (range: 41.39–56.60 cm^3^/m^2^). The lower limit of BSA-indexed pancreatic volume was 38 cm^3^/m^2^.

Both β-TM and β-TI patients were significantly younger than healthy subjects (*p* < 0.0001 and *p* = 0.005, respectively), while no gender difference was detected (*p* = 0.655 and *p* = 0.893, respectively).

**Figure 3:**
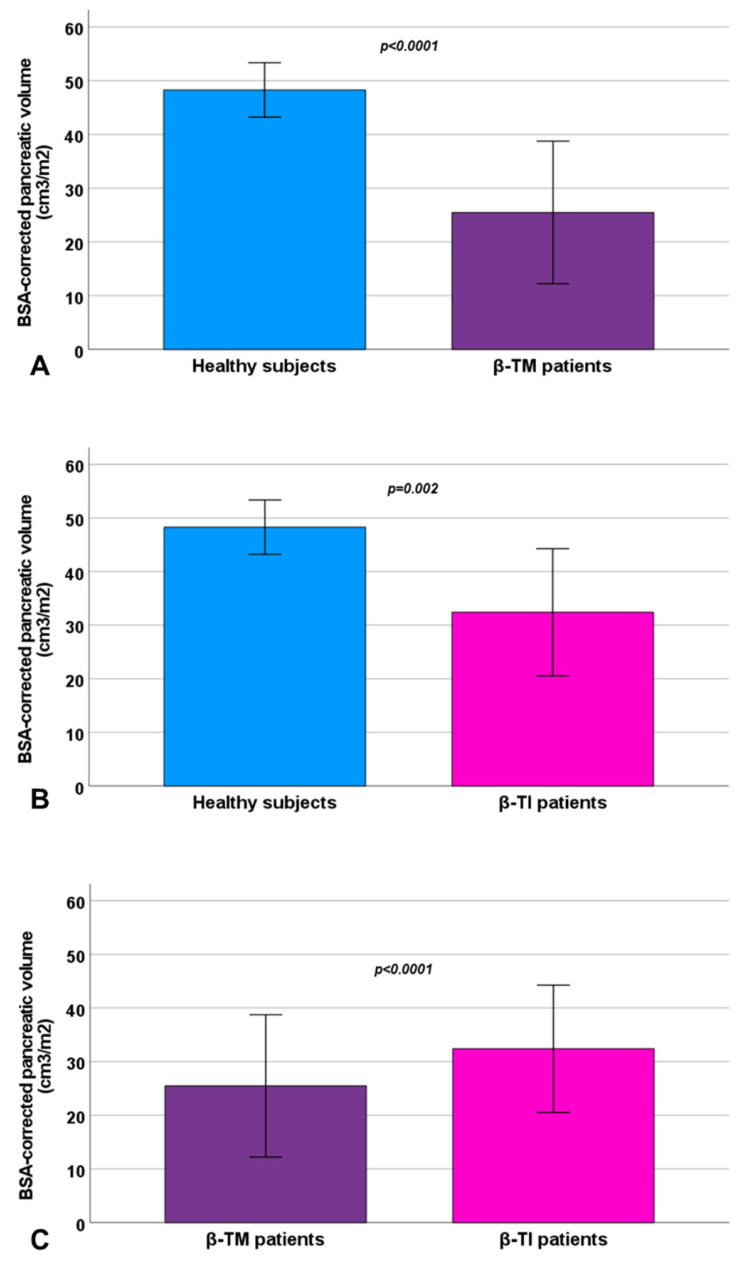
Comparison of BSA-corrected pancreatic volume between healthy subjects and β-TM patients (**A**), healthy subjects and β-TI patients (**B**), and β-TM and β-TI patients (**C**). The bars in the boxes represent the SD.

Compared to healthy subjects, both β-TI and β-TM patients exhibited a significantly decreased pancreatic volume (Figure 3A,B). The difference between β-TM and healthy subjects remained significant also after the correction for age (*p* < 0.0001).

### 3.4. Comparison Between β-TM and β-TI Patients

Table 1 shows the comparison between β-TI and β-TM patients. Age, sex, and frequency of splenectomy were comparable between the two groups. As per the definition, all β-TM patients were regularly transfused (> 4 transfusions per year) since early childhood and chelated. β-TI patients were less frequently regularly transfused and chelated and started regular transfusion and chelation therapies at a significantly higher age. Mean serum hemoglobin and ferritin levels and the frequency of alterations of glucose metabolism were significantly higher in β-TM patients. No difference was found in hepatic iron levels.

The β-TM group was characterized by significantly increased pancreatic iron levels and a significantly reduced pancreatic volume (Figure 3C).

When considering only the β-TI patients regularly transfused, the difference between the two patient groups remained significant for both pancreatic T2* values (11.85 ± 9.08 ms vs. 21.25 ± 8.99 ms, *p* = 0.001) and BSA-corrected pancreatic volume (25.48 ± 13.26 cm^3^/m^2^ vs. 38.64 ± 7.86 cm^3^/m^2^, *p* < 0.0001). In the ANCOVA model, the adjustment for the duration of regular transfusions, inversely correlated with both global pancreatic T2* values (R = −0.345; *p* = 0.001) and BSA-corrected pancreatic volume (R = −0.314; *p* = 0.003), did not alter the results (global pancreas T2* values: *p* = 0.050 and BSA-corrected pancreatic volume: *p* = 0.037).

### 3.5. Correlates and Determinants of Pancreatic Volume in β-TM

Table 2 shows the demographic, clinical, and MRI correlates of BSA-corrected pancreatic volume in β-TM patients. BSA-corrected pancreatic volume was comparable between males and females as well as between non-splenectomized and splenectomized β-TM patients, but it showed a weak inverse correlation with age and duration of transfusion therapy. BSA-corrected pancreatic volume was not correlated with serum levels of pre-transfusion hemoglobin and ferritin or MRI LIC values. A significant association was found between decreased pancreatic volume and increased pancreatic iron levels.

In β-TM patients, to detect the predictors of BSA-corrected pancreatic volume, a stepwise regression analysis was performed, including the model age, duration of regular transfusions, and global pancreas T2* values. Due to collinearity between age and duration of regular transfusions, each variable was paired with global pancreas T2* in two different models. In both models, global pancreas T2* values emerged as the only independent predictor of BSA-corrected pancreatic volume (standardized β coefficient = 0.229, *p* = 0.006).

The 90.8% of β-TM patients with a reduced pancreatic volume also had pancreatic iron overload. Patients with pancreatic iron overload were almost five times more likely to have a reduced pancreatic volume than patients without pancreatic iron overload (OR = 4.92, 95% CI = 1.44–16.77; *p* = 0.011).

### 3.6. Correlates and Determinants of Pancreatic Volume in β-TI

Table 2 shows the demographic, clinical, and MRI correlates of BSA-corrected pancreatic volume in β-TI patients. BSA-corrected pancreatic volume was not associated with age, gender, splenectomy, mean serum ferritin, and pancreatic iron levels. BSA-corrected pancreatic volume was directly correlated with serum hemoglobin levels and inversely correlated with MRI LIC values.

In β-TI patients, to detect the predictors of BSA-corrected pancreatic volume, a stepwise regression analysis was performed, including mean serum hemoglobin and MRI LIC values in the model. MRI LIC values emerged as the only independent predictor of BSA-corrected pancreatic volume (standardized β coefficient = −0.479, *p* = 0.038).

### 3.7. Pancreatic Volume and Glucose Metabolism in β-TM

Teen β-TM patients were already diagnosed with diabetes, and 106 performed the OGGT. Among the considered 116 patients, 81 (69.8%) had NGT, and 35 (30.2%) presented with disturbances of glucose metabolism. The prevalence of IFG, IGT, and DM in the considered β-TM population was, respectively, 5.2%, 8.6%, and 16.4%.

In non-diabetic patients, the mean FPG was 88.76 ± 10.31 mg/dL, and it was inversely correlated with BSA-corrected pancreatic volume (R = −0.234; *p* = 0.039).

**Figure 4:**
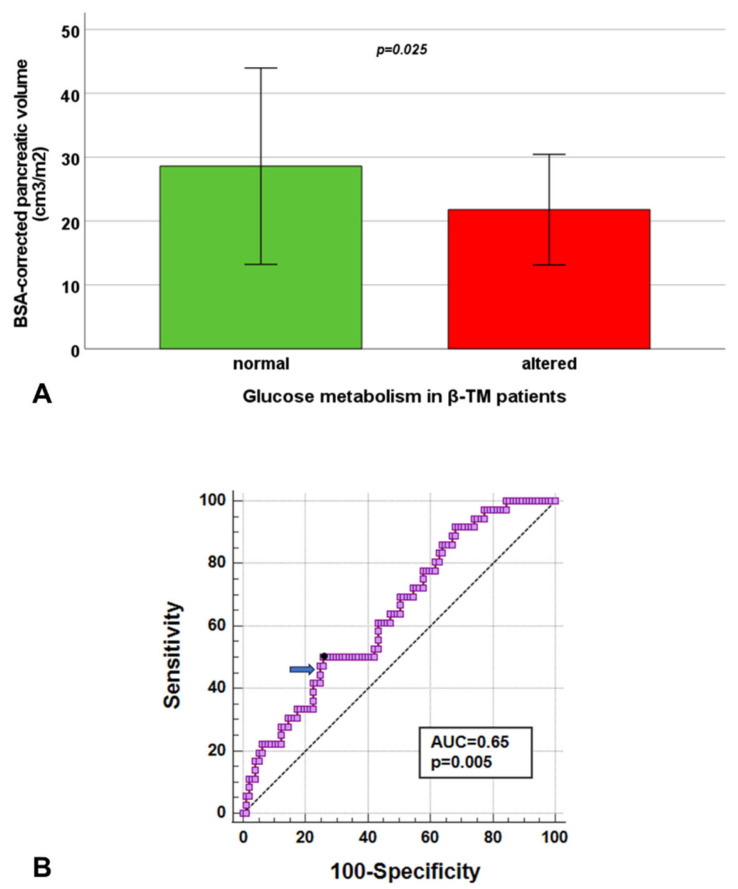
Association between BSA-corrected pancreatic volume and glucose metabolism in β-TM patients. (**A**) Mean BSA-corrected pancreatic volume in patients with normal and altered glucose metabolism. The bars in the boxes represent the SD. (**B**) ROC curve analysis of BSA-corrected pancreatic volume to predict the alterations of glucose metabolism. The arrow indicates the best cut-off based on Yuden J’s statistics.

β-TM patients with altered glucose metabolism showed a significantly lower BSA-corrected pancreatic volume than β-TM patients with normal glucose metabolism (21.79 ± 8.66 cm^3^/m^2^ vs. 28.59 ± 15.38 cm^3^/m^2^, *p* = 0.025) (Figure 4A). Out of the 35 patients with glucose metabolism disturbances, only one (2.9%) had a normal BSA-corrected pancreatic volume, while all presented with pancreatic iron overload. The 66.0% of the β-TM patients with a normal glucose metabolism had a reduced BSA-corrected pancreatic volume.

When compared to the healthy subjects, not only the β-TM group with alterations of glucose metabolism but also the β-TM group with normal glucose metabolism was characterized by a significantly lower BSA-corrected pancreatic volume (*p* < 0.0001 for both comparisons).

A significant difference between patients with and without altered glucose metabolism was also found in pancreatic T2* values (6.40 ± 3.46 ms vs. 13.07 ± 8.89 ms, *p* < 0.0001), while MRI LIC values were comparable between the two groups (7.23 ± 6.19 mg/g dw vs. 6.50 ± 11.66 mg/g dw, *p* = 0.068).

At ROC curve analysis, a BSA-corrected pancreatic volume ≤ 20.48 cm^3^/m^2^ predicted the presence of abnormal glucose metabolism with a sensitivity of 51.4% and a specificity of 72.8% (*p* = 0.018). The AUC was 0.63 (95% CI = 0.54–0.72) (Figure 4B). A pancreatic T2* ≤ 8.4 ms predicted the presence of abnormal glucose metabolism with a sensitivity of 85.7% and a specificity of 60.5% (*p* < 0.0001). The AUC was 0.73 (95% CI = 0.64–0.81). The Delong’s test did not show a significant difference among the AUCs (*p* = 0.066).

Based on the identified cut-offs, three groups of patients were identified: group zero with both BSA-corrected pancreatic volume and pancreas T2* higher than the identified cut-offs, group one with only the BSA-corrected pancreatic volume or the pancreas T2* below the identified cut-offs, and group two with both BSA-corrected pancreatic volume and pancreas T2* below the identified cut-offs. The frequency of alteration of glucose metabolism was 7.5% in group one, 30.2% in group two, and 59.3% in group three (*p* < 0.0001). OR for abnormal glucose metabolism was 5.29 (95% CI = 1.63–17.17; *p* = 0.005) for group one versus group zero, 17.82 (95% CI = 4.97–63.83; *p* < 0.0001) for group two versus group zero, and 3.64 (95% CI = 1.28–8.84; *p* = 0.014) for group two versus group one.

### 3.8. Pancreatic Volume and Glucose Metabolism in β-TI

Out of the 19 β-TI patients, none were already diagnosed with diabetes, and the OGTT was performed in 17 (89.5%) of them. An IFG was diagnosed in one patient, who showed a decreased BSA-corrected pancreatic volume and pancreatic iron overload.

The mean FPG was 79.73 ± 14.33 mg/dL, and it was not correlated with BSA-corrected pancreatic volume (R = 0.227, *p* = 0.415).

## 4. Discussion

We measured pancreatic volume in β-thalassemia patients by MRI using planimetry, and we assessed its clinical correlates.

Our study demonstrated for the first time that, independently from the presence of alterations in glucose metabolism, β-thalassemia is associated with a reduction in pancreatic volume compared to the healthy population. The most likely explanations for these findings are the decreased endogenous insulin secretion and the chronic inflammation, determined by the pancreatic iron accumulation. In β-thalassemia patients, excess iron saturates the ability of the transferrin iron transport system, resulting in the circulation of non-transferrin bound iron (NTBI) and labile plasma iron (LPI) in the plasma, which then accumulate in vulnerable cells [45,46]. Free iron, even in minimal quantities, is highly reactive and can induce oxidative stress, generating significant amounts of reactive oxygen species (ROS) [46,47,48]. This leads to further ROS-induced damage to DNA and proteins, reduced insulin synthesis and secretion, and increased apoptosis [21,49]. Iron can also lead to beta-cell dysfunction and death through ferroptosis, a form of non-apoptotic cell death triggered by iron accumulation. It has been postulated that, following the death of pancreatic cells due to the cytotoxic effects of iron, there may be a gradual replacement of the pancreatic tissue with fat [50]. Indeed, pancreatic fatty replacement was demonstrated to be highly frequent among patients with β-TM [51,52]. Pancreatic fat infiltration in and around islets is associated with impaired beta cell dysfunction [53,54,55,56]. The islets of Langerhans constitute 1–2% of the total pancreatic volume, but the hormones produced, above all insulin, serve as trophic factors in the exocrine pancreas [57,58]. Moreover, excessive ROS production triggers an inflammatory response through the recruiting of circulating inflammatory cells and fibroblast progenitors and the overactivation of the nuclear enzyme poly (ADP-ribose) polymerase, which promotes the expression of inflammatory mediators [59]. The chronic inflammation can be further exacerbated by repeated exposure to foreign antigens from transfusions [60]. An autoptic study conducted in non-thalassemic patients with diabetes demonstrated an association between lymphocyte infiltration and pancreatic exocrine atrophy [61].

The comparison between β-TM and β-TI patients demonstrated an increased pancreatic atrophy in the β-TM group, which is most likely the consequence of the increased pancreatic siderosis. The differences in pancreatic iron levels and volume between the regularly transfused β-TI and β-TM patients persisted even after adjusting for the duration of regular transfusions. Therefore, although the differences in transfusional burdens and durations explain much of the disparity in extra-hepatic iron levels between transfused β-TI and β-TM patients, they cannot be considered the only cause. A role may be played by different levels of hepcidin, a key regulator of iron homeostasis [62,63,64], expressed not only in the liver but also in the pancreatic islets [65]. Transfusional iron overload is characterized by increased levels of hepcidin, so hepcidin has been proposed as a simple potential marker of iron overload in β-TM [66].

In both β-TM and β-TI groups, the pancreatic volume was significantly higher in males than in females, in line with anatomical and imaging studies on PV [67,68,69,70]. However, upon normalization to BSA, the distinction between sexes was no longer significant, supporting that indexing pancreatic dimensions according to BSA can compensate for the sex-related differences in body size and provide a standardization useful for clinical practice. In β-TM, we found a weak inverse association between pancreatic volume and aging. In healthy subjects, the pancreatic volume was demonstrated to increase during childhood, to reach a plateau between 20 and 50 years, and to decline thereafter [67,68,70]. In β-TM, characterized by accelerated aging [71], the age-related decline of pancreatic volume can start early.

In β-TM, pancreatic iron levels emerged as the strongest predictor of BSA-corrected pancreatic volume, with pancreatic iron overload conveying a five times increased risk of a reduced pancreatic volume. The discrepancy with the study by Au et al., which did not detect an association between pancreatic volume and iron in a cohort of 72 β-TM patients [34], can be due to the smaller sample size and the different ethnicity.

In β-TI, hepatic iron levels emerged as the strongest predictor of BSA-corrected pancreatic volume. Conversely, pancreatic iron and volume were not correlated, likely because numerous patients exhibited normal or only mildly reduced values of these parameters. A study involving more β-TI patients may be useful to assess if the demographic and clinical correlates of pancreatic volume differed between non-transfused and regularly transfused patients, as already demonstrated for pancreatic T2* values [15].

Due to the relatively low number of β-TM patients with OGGT data, all the alterations of glucose metabolism were considered together and were demonstrated to be associated with a reduction in BSA-corrected pancreatic volume. A BSA-corrected pancreatic volume ≤ 20.48 cm^3^/m^2^ emerged as the best cut-off for predicting an abnormal glucose metabolism. The moderate sensitivity can be explained by the fact that glucose dysregulation can be influenced by genetic, environmental, and behavioral factors [72]. Importantly, there was no difference in the diagnostic ability of pancreatic volume and iron for alteration of glucose metabolism, and the presence of both BSA-corrected pancreatic volume and pancreas T2* below the identified cut-offs was associated with a significantly increased risk for an altered OGGT than the presence of only one of these parameters below the detected cut-off. Therefore, although additional cross-sectional and prognostic studies are needed to confirm our data, the combined use of both pancreatic volume and iron levels may enable the identification of three categories of patients with low, intermediate, and high risk for alterations of glucose metabolism. In high-risk patients, close monitoring and the intensification or adjustment of iron chelation therapy may help prevent the development of manifest clinical disease.

The cross-sectional design of our study, which assessed correlations at a single time point, likely hindered the identification of a significant association between alterations in glucose metabolism and hepatic iron levels. Dysregulation of glucose metabolism does not typically occur as an immediate consequence of iron deposition and may not be reversible. At the time of assessment, chelation therapy may have successfully reduced hepatic iron levels, while the reduction in pancreatic iron has been shown to be a slow and difficult process [73].

In the β-TI group, the presence of only one patient with altered glucose metabolism prevented the study of its association with pancreatic volume.

### Limitations

This study used a cross-sectional design, limiting our ability to determine cause-and-effect relationships. Longitudinal studies are needed to clarify whether changes in pancreatic volume contribute to or result from the development of alterations in glucose metabolism.

For the β-TI group, the small sample size affected the statistical power, making it more challenging to identify meaningful patterns or significant differences.

We had data about the baseline insulin for a limited number of patients, which prevented to determine the homeostasis model assessment of β-cell function (HOMA-β) and of insulin resistance (HOMA-IR) indexes. Although these indexes can provide valuable insights, they rely on fasting glucose and insulin levels and may mask the truly impaired insulin release and resistance. Additionally, in thalassemia patients, insulin sensitivity can be impaired even in cases that appear normoglycemic [74].

We did not measure the glycosylated hemoglobin A1c (HbA1c), which usually serves as a crucial biomarker for assessing long-term glycemic control, representing both fasting and postprandial plasma glucose levels over the past 2–3 months. However, in conditions affecting hemoglobin levels or red blood cell turnover rates, such as thalassemia, HbA1C is not considered a suitable marker for retrospective glycemic assessment [75].

## 5. Conclusions

β-thalassemia syndromes are characterized by a reduction in the pancreatic volume, which is more pronounced among β-TM patients. The strongest predictor of BSA-corrected pancreatic volume is represented by pancreatic iron levels in β-TM and hepatic iron levels in β-TI. The combined evaluation of the pancreatic volume and iron levels may allow for the stratification of β-TM patients into three distinct risk categories for potential dysregulation of glucose metabolism: low, intermediate, and high.

## Figures and Tables

**Table 1:** Comparison of demographic, clinical, and MRI data between patients with beta-thalassemia major and intermedia.

	β-TM Group (*N* = 145)	β-TI Group (*N* = 19)	*p*-Value
Age (years)	38.16 ± 10.08	39.63 ± 12.67	0.470
Females, *N* (%)	83 (57.2)	10 (52.6)	0.703
Splenectomy, *N* (%)	63 (43.4)	12 (63.2)	0.105
Regular transfusions, *N* (%)	145 (100)	11 (57.9)	<0.0001
Age at start of regular transfusions (years)	1.06 ± 0.82	23.08 ± 21.24	<0.0001
Duration of regular transfusions (years)	35.90 ± 10.55	20.61 ± 18.00	0.036
Patients in chelation therapy, *N* (%)	145 (100)	15 (78.9)	<0.0001
Age at start of chelation therapy (years)	5.34 ± 7.35	27.83 ± 19.01	0.001
Mean pre-transfusion hemoglobin (g/dL)	9.55 ± 0.51	8.85 ± 1.20	0.018
Mean serum ferritin (ng/mL)	1012.63 ± 865.07	615.75 ± 541.77	0.018
Altered glucose metabolism, *N* (%)	35/116 (30.2)	1/17 (5.9)	0.040
MRI LIC (mg/g dw)	6.12 ± 9.47	5.39 ± 6.34	0.522
Global pancreas T2* (ms)	11.86 ± 9.08	23.78 ± 9.69	<0.0001
Pancreatic iron overload, *N* (%)	130 (89.7)	10 (52.6)	<0.0001
BSA-indexed pancreatic volume (cm^3^/m^2^)	25.48 ± 13.26	32.40 ± 11.87	0.007
Reduced pancreatic volume, *N* (%)	10 (52.6)	130 (89.7)	<0.0001

TM = thalassemia major, TI = thalassemia intermedia, *N* = number, MRI = magnetic resonance imaging, LIC = liver iron concentration, BSA = body surface area.

**Table 2:** Demographic, clinical, and MRI correlates of BSA-corrected pancreatic volume in patients with beta-thalassemia major and with beta-thalassemia intermedia.

	β-TM Group	β-TI Group
*Categorical Variables*	*Difference of BSA-Corrected Pancreatic Volume Between Two Groups (Absent vs. Present)*	
Females	27.55 ± 14.94 cm^3^/m^2^ vs. 23.94 ± 11.72 cm^3^/m^2^ (*p* = 0.197)	36.63 ± 11.73 cm^3^/m^2^ vs. 28.60 ± 11.21 cm^3^/m^2^ (*p* = 0.146)
Splenectomy	24.37 ± 11.42 cm^3^/m^2^ vs. 26.92 ± 15.31 cm^3^/m^2^ (*p* = 0.441)	29.39 ± 11.53 cm^3^/m^2^ vs. 34.16 ± 12.21 cm^3^/m^2^ (*p* = 0.414)
Pancreatic iron overload	36.57 ± 19.77 cm^3^/m^2^ vs. 24.20 ± 11.74 cm^3^/m^2^ (*p* = 0.004)	35.13 ± 11.21 cm^3^/m^2^ vs. 29.37 ± 12.49 cm^3^/m^2^ (*p* = 0.304)
*Continuous Variables*	*Correlation (R, p-Value) with BSA-Corrected Pancreatic Volume*	
Age	R = −0.268, *p* = 0.001	R = 0.285, *p* = 0.237
Duration of regular transfusions (for regularly transfused patients)	R = −0.288, *p* = 0.008	R = −0.314, *p* = 0.544
Mean serum hemoglobin	R = −0.132, *p* = 0.198	R = 0.577, *p* = 0.019
Mean serum ferritin	R = −0.056, *p* = 0.585	R = −0.297, *p* = 0.264
MRI LIC	R = 0.067, *p* = 0.429	R = −0.635, *p* = 0.003
Global pancreas T2*	R = 0.221, *p* = 0.008	R = 0.296, *p* = 0.218

TM = thalassemia major, TI = thalassemia intermedia, BSA = body surface area, MRI = magnetic resonance imaging, LIC = liver iron concentration.

## Data Availability

The data presented in this study are available on request from the corresponding author. The data are not publicly available due to privacy.
